# Commentary on "*Systematic review about complementary medical hyperthermia in oncology*" by Liebl et al.

**DOI:** 10.1007/s10238-022-00902-4

**Published:** 2022-10-14

**Authors:** Elisabeth Arrojo, Giammaria Fiorentini, Pirus Ghadjar, Carrie Minnaar, A. Marcell Szasz, Andras Szasz

**Affiliations:** 1 University Hospital Marques de Valdecilla, Santender, Cantabria Spain; 2 Medical Institute of Advanced Oncology (INMOA), Madrid, Spain; 3 Former Director Medical Oncology Unit and Hyperthermia Service, Onco-Hematology Department, Azienda Ospedaliera Marche Nord, Pesaro, Italy; 4 Department of Radiation Oncology, Charité – Universitätsmedizin Berlin, Freie Universität Berlin and Humboldt-Universität Zu Berlin, Berlin, Germany; 5 Department of Radiation Sciences, University of the Witwatersrand, Johannesburg, South Africa; 6 Wits Donald Gordon Academic Hospital, Johannesburg, South Africa; 7 Division of Oncology, Department of Internal Medicine and Oncology, Semmelweis University, Budapest, Hungary; 8 Biotechnics Department, Hungarian University of Agriculture and Life Sciences, Godollo, Hungary

We read the recent article by Liebl et al. [1]. Unfortunately, several important critical points should be brought to the readers' attention.

A variety of hyperthermia methods exist and each has fundamental differences in actions and effects. The authors discuss "complementary hyperthermia" and discriminately include only electrohyperthermia and whole-body hyperthermia (WBH) in this category. This is despite the appropriate definitions for methods of heating used in the field of oncologic hyperthermia having been described [2]. The selection of articles is not inclusive leading to a biased interpretation of the results. There are several positive phase III trials for capacitive hyperthermia (see Table 1), underscoring the authors' incorrect assessment of hyperthermia techniques.

**Table 1:** The authors did not take the following clinical studies with EH methods into account

Malignancy	*n*	Intervention	Arms	References	Remark
Uterus cervix	40	RT ± cHT	2	[1]	RR + ST
Uterus cervix	110	RT ± cHT	2	[2]	RR + ST
Uterus cervix	271	RT + ChT ± mEHT	2^a^	[3]	RR + ST
Non-small-cell lung cancer	80	RT ± cHT	2	[4]	RR + ST
Non-small-cell lung cancer	97	ChT ± mEHT ± IVC	2	[5]^d^	ST + QoL
Head and neck cancer	65	RT ± cHT	2	[6]	RR
Head and neck cancer	56	RT ± cHT	2	[7]	RR + ST
Esophagus cancer	66	RT + ChT ± cHT	2	[8]	RR + ST
Esophagus cancer	40	ChT ± cHT	2	[9]	RR
Bone metastases	57	RT ± cHT	2	[10]	RR
Uterus cancer	38	ChT ± mEHT	2	[11]	RR + ST
Urinary bladder cancer	49	RT ± cHT	2	[12]	RR + ST
Peritoneal carcinomatosis	260	IPCh ± mEHT ± TCM	2	[13]^d^	RR
Nefopam pharmacokinetics	12^c^	ChT ± mEHT	2	[14]	PhK
Fentanyl pharmacokinetics	12^c^	ChT ± mEHT	2	[15]	PhK
Non-small-cell lung cancer	19	RT + cHT	1 + ^b^	[16]	RR + ST
Non-small-cell lung cancer	35	RT + cHT	1	[17]	ST
Gastric cancer	21	RT + cHT	1	[18]	RR + ST
Rectal cancer	81	RT + ChT + cHT	1	[19]	RR
Rectal cancer	76	RT + ChT + mEHT	1	[20]	RR + AE
Rectal cancer	120	RT + ChT + mEHT	1	[21]	RR + ST
Pediatric brain tumors	41	IT + mEHT	1	[22]	ST
Soft tissue sarcoma	27	RT + cHT	1	[23]	RR
Recurrent breast cancer	26	RT + cHT	1	[24]	RR
Brain malignancies	140	ChT ± mEHT	1	[25]^d^	RR + ST
Non-small-cell lung cancer	15	ChT ± mEHT	1	[26]^d^	DE
Glioblastoma	24	ChT ± mEHT	1	[27]^d^	DE

*ChT* chemotherapy, *RT* radiotherapy, *cHT* capacitively coupled hyperthermia, *mEHT* modulated electrohyperthermia, *RR* response rate, *ST* survival time, *PhK* pharmacokinetics, *AE* adverse effects, *IPCh* intraperitoneal chemotherapy, *TCM* Traditional Chinese Medication, *QoL* the quality of life, *IVC* intravenous vitamin C, *DE* dose escalation

^a^Additional sub-arm for HIV patients

^b^Historical reference arm;

^c^Healthy, voluntary participants;

^d^Falsely interpreted

Our major points are:

A. The methodologies and techniques are not correctly described, leading to inaccurate definitions that are not used in the field, and are therefore not useful for the readers.
B. The authors have missed essential articles, which may be related to their crude methodology, definitions, and the discriminate selection method.
C. The article Liebl et al. [1] contains several errors and biases:
  1. The article only evaluates WBH and capacitive coupled hyperthermia, and this selection does not meet the criteria for a "systematic review." Many applications (such as phased-array, RF radiative heating, nano-heating, and Japanese capacitive hyperthermia) are also techniques employed in the field of complimentary hyperthermia but have been systematically neglected.
  2. Contrary to the title of the text of the article;
    - The selection method described in the text is misleading for the readers: *"… we have included in this review only hyperthermia methods that do not belong to conventional medicine and titled these alternative methods*." Hyperthermia methods are mostly applied when other treatments alone do not provide satisfactory results. Hyperthermia is a complementary treatment, employed to compliment or enhance the efficacy of conventional therapies. Hyperthermia is not an alternative treatment
    - The referred methods have medically accepted and significant Phase III trial results, yet the authors claim that these "*do not belong to conventional medicine*." What is the definition of conventional medicine, according to the authors, and on what basis can they make such a claim when these results have been accepted in peer-reviewed journals?
  3. The authors do not define electrohyperthermia (EH). It is likely that the authors mean "*capacitive coupling*," and however, the authors have also included inductive heating results and discussed them in detail (Loboda et al. cite ref. {54}). Inductive heating refers to the use of electromagnetism and magnetic fields and does not include capacitive heating.
  4. Authors have further particularities in their selection of studies to include in the systemic review. It is not clear how the selection excludes the following:
    - *"…125 studies did not use alternative hyperthermia"*. But all selected hyperthermia applications in the article are complementary to “conventional” (chemotherapy/radiotherapy) medicine and are not an alternative to “conventional” medicine.
    - *"…43 studies, multiple interventions were administered simultaneously."* But almost all hyperthermia techniques, including those in the article, are applied complementary to other treatments and are therefore applied simultaneously with other therapies (mostly with chemotherapy).
    - *"…assessment of hyperthermia was not possible."* Authors do not define how they measured the criteria of *"assessment"* in the selection.
  5. The tables in the article combine the WBH and the local EH results. However, these techniques are fundamentally different, in their methods, indications, safety limits, and physiological actions, and can therefore not be compared directly or be discussed using the same criteria for evaluation.
  6. Some statements lack the full information from the article that is referenced and this provides a negative or biased view. For example, when referring to the study on brain tumors by Fiorentini et al., the following statement is made: “*Adverse events caused by EH in the RCT by Fiorentini *et al*. {56} included headache, scalp burn and seizures. More than an hour after treatment, seizures occurred in 4 additional patients*.” The authors fail to mention that the study is on brain tumors, and that indeed tumors themselves cause seizures and headaches and that it is not possible to confirm that the adverse events are from the hyperthermia treatments and not from the advanced stage of disease or the concurrent treatments.
  7. The authors claim that only the adverse effects of the studies with multiple interventions are reported due to the difficulty in confirming the benefit of the hyperthermia when multiple interventions are administered. The same should therefore be true of the adverse events and toxicity. This is selective reporting of the negative effects of 43 trials without considering the benefits.
  8. The article ignored numerous phase II and phase III clinical trials investigating capacitive coupling (electrohyperthermia) which reported significant improvement in the local response and survival times (Table 1.) Many of the ignored studies fall into the “first level category" of evidence.
  9. The entire evaluation does not correctly categorize the clinical phases of the trials. It mixes the phase 1 (safety trial), phase 2 (efficacy trial), phase 3 (clinical benefits approval), and phase 4 (market surveillance) methods, where the goals of the studies are obviously different, and so their evaluation has to differ as well.
  10. The category of the first level evidence {Fig. 2.} uses category "*2b-*," which does not exist in the Oxford evidence rank. Furthermore, Fig. 2 evaluates the phase III study as 2b evidence. According to the Oxford evidence scale, the prospective randomized phase III study is 1b. 2b is a retrospective study, which has entirely different conditions.
  11. Authors use different and unclear categories which have no conventional meaning and no explanations. Some points:
    - What is the difference between the "*single-arm*" and the "*cohort study*"? The single-arm study must also use a cohort; otherwise, it is only a case series.
    - Authors should provide a better explanation for the difference between the "*multiple intervention*" category (Table 7) and the other categories (for example, the radio-chemo-thermo categories), in previous tables.
    - The scoring system in Table 3 is undefined (What is the Berlin scoring system?).
    - The article misinterprets the aim of the clinical results in the article by Kim et. al. {58}. The clinical study used ~ 20% less radiotherapy in the active arm and had similar results to the larger radiation dose in the control group. It is an important and clinically positive result, however, its interpretation in the article is negative.
    - The research papers by Minnaar et al. {52} and {53} are shown as published in 2019, while these were in 2020. The explanation of outcomes uses study arms A and B, but it is not identified which arm is the active and passive.
    - The trials of Douwes et al. {79}, Gadaleta-Caldarola et al. {80}, Yoo et al. {82} are phase 2 retrospective trials, with evidence level 2b, so these are in the wrong place and belong to Fig. 2. and Table 3.
    - The evaluation of Yoo et al. {82} has the expression "*time to death*," the meaning of which is not clear. Is it overall survival (time from the first diagnosis), or survival from the first hyperthermia treatment, or other? This study had a successful safety (dose escalation) phase but was not registered in Table 4.
    - The studies by Ko et al. {115} and Qiao et al. {124} was identified as a "*cohort study*" but were applied to "*different entities of cancer*." How may we understand the category "*cohort*"?
    - The phase 2 randomized prospective clinical trials of Ou et al. {122} (1b evidence), Pang et al. {123} (1b evidence) and Fiorentini et al. {102} are retrospective double-arm studies (2b evidence) in Table 6., despite the fact that the others listed here (40 studies) are single-arm studies or case reports. These trials are missing from Table 3. efficacy studies.
    - The application of some heating techniques in a palliative setting where there is no cure possible and patients have failed all other treatments is not discussed. In these studies, the heating technique is applied without any chemotherapy or radiotherapy (for example, Fiorentini et al. {102}).

When considering the criticisms of individual studies, it is clear that the authors have either not understood the methodology of the studies. Unfortunately, this comes across as an attempt to discredit some studies by using only selective information. The interpretation and discussion should be reviewed and reassessed in order to prevent what could be perceived to be a biased interpretation of the results. For example, regarding the phase 3 clinical studies by Minnaar et al., the following statements are made, and when reviewing the articles, the answers to all of these questions can be found:

1. “*No data on the target temperature in the tumor field are reported.”* The reason for the lack of temperature measurement and the dosing methods is discussed in detail {52}.
2. *“In these studies, many calculations are performed. However, in the exact comparison of the intervention and control group regarding the therapy, these data are missing. Therefore, it is not possible to accurately compare the treatments between the two arms with and without hyperthermia.”* There are numerous exact comparisons between the active (hyperthermia) and control arms. In fact, the objective of all three papers is to compare the hyperthermia arm to the non-hyperthermia arm, and therefore, all of the calculations are direct comparisons, including frequency tables with chi-squared and Fischer exact tests, multivariate regression analyses, and paired and unpaired t-tests evaluating local disease control, disease-free survival, toxicity, and quality of life between the two groups. All calculations are described in the methodology, reported in results and discussed in the discussion.
3. *“In addition, information about prior treatments is not specified and a description of possible additional co-interventions is missing.”* Prior treatment to cervical cancer is an exclusion, and the investigation is into the primary management of locally advanced cervical cancer, there are therefore no related prior treatments to specify. Additionally, there are no co-interventions, and the prescribed treatments are described in detail and include only radiotherapy, brachytherapy and cisplatin — the standard of care recognized internationally. There are no other standard/accepted interventions for locally advanced cervical cancer. This statement is therefore redundant.
4. *“For the endpoints tumor response and local disease control, reasons for the drop-out of part of the participants are not given. Therefore, it cannot be excluded that for these endpoints only suitable patients were considered… The reasons for the missing data of part of the participants are not stated; therefore, selective reporting cannot be excluded. Additionally, with such a high drop-out rate and without any reasons given, the comparability of the groups cannot further be assumed. It is therefore possible that healthier or more motivated patients remained in the study. Those patients then may achieve a better result and do not constitute a representative sample*.” In all three papers, the CONSORT diagrams give the reasons for the drop-outs. The dropout rate was not considered to be abnormally high (4.7% in the control group and 2.9% in the intervention group).

The systemic review and the conclusions derived by the authors are flawed due to (a) methodological errors in selection and interpretation of papers (b) incorrect interpretation of the technology for hyperthermia delivery and (c) excluding key articles from their systematic review as detailed above. These could result in the erroneous view of hyperthermia to the readers, thereby depriving patients of a multifaceted therapeutic modality that has been shown to be effective when used with radiotherapy and/or chemotherapy for a wide range of malignancies.
